# Supplementary material for: EnzML: multi-label prediction of enzyme classes using InterPro signatures
Source: BMC Bioinformatics. 2012 Apr 25;13:61. doi: 10.1186/1471-2105-13-61 (PMC3483700; doi:10.1186/1471-2105-13-61)
Supplement: Addtional file 5 — The Java code to format the data files, evaluate and predict. The file enzml_java_code.tar.gz contains the Java code used to format database data to ARFF and XML formats, to execute cross and train-test (jackknife) evaluations and to record evaluation results to database. More information is included in the readme.txt file and the Javadoc files. The code can be used with a MySQL database. To use a different database software, other JDBC drivers might be required. [file 1471-2105-13-61-S5.gz › java_code/enzml2011/doc/uk/ac/ed/inf/enzml/mulan/learn/database/MulanDbManager.html]

MulanDbManager


---


|  |  |  |
| --- | --- | --- |
| **Overview** \| **Package** \| **Class** \| **Use** \| **Tree** \| **Deprecated** \| **Index** \| **Help** | |  |
| **PREV CLASS**   **NEXT CLASS** | **FRAMES**    **NO FRAMES**     **All Classes** |  |
| SUMMARY: NESTED \| FIELD \| CONSTR \| METHOD | DETAIL: FIELD \| CONSTR \| METHOD |  |


---


## uk.ac.ed.inf.enzml.mulan.learn.database Class MulanDbManager

```
java.lang.Object
  uk.ac.ed.inf.utils.database.Manager
      uk.ac.ed.inf.utils.database.DbManager
          uk.ac.ed.inf.enzml.mulan.learn.database.MulanDbManager
```

---

``` public class MulanDbManager extends uk.ac.ed.inf.utils.database.DbManager ```

The database manager controls the writing to database. It provides objects (
`MulanDbCreator`, `MulanDbWriter`, `MulanDbReader`) to
write to database the results (evaluation) of Mulan multi-label machine
learning.
The database connection properties are read from a .props text file. A
mulan\_database.props file example for MySql is in the Java source.

**Version:**
:   9 Jun 2010

**Author:**
:   Luna De Ferrari luna.deferrari-at-ed.ac.uk

---

**Field Summary**

**Fields inherited from class uk.ac.ed.inf.utils.database.DbManager**

`m_dbWriter`


**Constructor Summary**

`MulanDbManager(java.lang.String dbConnPath)`
  
          The `DbManager` constructor creates `DbCreator`,
`DbWriter` and `DbReader` objects and writes the necessary
tables to database.


**Method Summary**

**Methods inherited from class uk.ac.ed.inf.utils.database.DbManager**

`closeConnection, connectionIsValid, getDatabaseName, getDbConnection, getDbCreator, getDbReader, getDbWriter, getTableManager, getTableManager, refreshConnection, runAnalyzeTable`

**Methods inherited from class java.lang.Object**

`equals, getClass, hashCode, notify, notifyAll, toString, wait, wait, wait`

**Constructor Detail**

### MulanDbManager

```
public MulanDbManager(java.lang.String dbConnPath)
```

:   The `DbManager` constructor creates `DbCreator`,
    `DbWriter` and `DbReader` objects and writes the necessary
    tables to database.

    **Parameters:**: `dbConnPath` -


---


|  |  |  |
| --- | --- | --- |
| **Overview** \| **Package** \| **Class** \| **Use** \| **Tree** \| **Deprecated** \| **Index** \| **Help** | |  |
| **PREV CLASS**   **NEXT CLASS** | **FRAMES**    **NO FRAMES**     **All Classes** |  |
| SUMMARY: NESTED \| FIELD \| CONSTR \| METHOD | DETAIL: FIELD \| CONSTR \| METHOD |  |


---
